# Infection-prevention and control interventions to reduce colonisation and infection of intensive care unit-acquired carbapenem-resistant *Klebsiella pneumoniae*: a 4-year quasi-experimental before-and-after study

**DOI:** 10.1186/s13756-018-0453-7

**Published:** 2019-01-10

**Authors:** Meiling Li, Xiaoli Wang, Jiahui Wang, Ruoming Tan, Jingyong Sun, Lei Li, Jie Huang, Jun Wu, Qiuying Gu, Yujin Zhao, Jialin Liu, Hongping Qu

**Affiliations:** 1 Department of Critical Care Medicine, Ruijin Hospital, Shanghai Jiao Tong University School of Medicine, No.197 Ruijin ER Road, Shanghai, 200025 China; 2 Department of Clinical Microbiology, Ruijin Hospital, Shanghai Jiao Tong University School of Medicine, No.197 Ruijin ER Road, Shanghai, 200025 China

**Keywords:** Carbapenem-resistant *Klebsiella pneumoniae*, Infection-prevention and control intervention, De-escalation, Incidence, Catheter-related infection, intensive care unit

## Abstract

**Objective:**

To determine whether infection-prevention and control (IPC) interventions can reduce the colonisation and infection of intensive care unit (ICU)-acquired carbapenem-resistant *Klebsiella pneumoniae* (CRKP) in a general ICU ward in China.

**Methods:**

We used a quasi-experimental before-and-after study design. The study was conducted in 4 stages: baseline period, January 2013–June 2013; IPC interventions period including de-escalation and targeted bundle interventions, July 2013–June 2014; modified IPC interventions period, July 2014–June 2015; and follow-up period, July 2015–June 2016. We used modified de-escalation interventions according to patient-risk assessments to prevent the transmission of CRKP.

**Results:**

A total of 629 patients were enrolled in study. The incidence of ICU-acquired CRKP colonisation/infection was 10.08 (4.43–16.43) per 1000 ICU patient-days during the baseline period, and significantly decreased early during the IPC interventions, but the colonisation/infections reappeared in April 2014. During the modified IPC intervention and follow-up periods, the incidence of ICU-acquired CRKP colonisations/infections reduced to 5.62 (0.69–6.34) and 2.84 (2.80–2.89), respectively, with ongoing admission of cases with previously acquired CRKP. The incidence of ICU-acquired CRKP catheter-related bloodstream infections decreased from 2.54 during the baseline period to 0.41 during the follow-up period. The incidence of ventilator-associated pneumonia and skin and soft tissue infections showed a downward trend from 2.84 to 0.41 and from 3.4 to 0.47, respectively, with slight fluctuations.

**Conclusions:**

Comprehensive IPC interventions including de-escalation and targeted bundle interventions showed a significant reduction in ICU-acquired CRKP colonisations/infections, despite ongoing admission of patients colonised/infected with CRKP.

**Electronic supplementary material:**

The online version of this article (10.1186/s13756-018-0453-7) contains supplementary material, which is available to authorized users.

## Introduction

*Klebsiella pneumoniae* infections are a serious contemporary problem in intensive care units (ICUs) worldwide and primarily affect critical and immunocompromised patients [1]. Carbapenems are the last class of β-lactam drugs that have retained their anti-Gram-negative activity; however, multidrug-resistant (MDR) bacteria, especially carbapenem-resistant *Klebsiella pneumoniae* (CRKP), have emerged and disseminated worldwide [2, 3]. In our hospital, the incidence of CRKP has increased remarkably from 7.3% in 2011 to 25% in 2015 (unpublished). Most of CRKP strains exhibit multidrug resistance and fail to respond to conventional therapy, which has resulted in a 28-day attributable mortality rate of 30–70% [4]. In 2013, global agencies such as the Centers for Disease Control and Prevention declared carbapenem-resistant *Enterobacteriaceae* spp. as an immediate public health threat that required urgent and aggressive action [5].

Promiscuous plasmids and clonal outbreaks exacerbate the worldwide spread of CRKP [6]. Risk factors associated with the acquisition of CRKP bacteria include poor functional status, comorbidities, ICU stay, use of invasive devices, immunosuppression, and exposure to multiple antibiotics before the initial culture [7]. Patient-to-patient cross-transmission and hand carriage by health care workers are the main modes of spread of CRKP [8, 9]. In addition to the antimicrobial stewardship programs, coordinated infection-prevention and control (IPC) interventions such as contact precautions, hand hygiene, and active surveillance of patients at risk for CRKP carriage and infection are advocated for effectively stopping the spread of CRKP outbreaks. Robust evidence for IPC interventions effectiveness in individual studies is limited in developed countries [10–12]. Additionally, the interventions vary widely by region and effect. Moreover, multifaceted implementation programs in low-income and middle-income countries, especially in hospital settings with restricted resources, are inconclusive.

This retrospective study aimed to evaluate the epidemiologic characteristics of CRKP in the general ICU during July 2013–June 2016 and assess the effect of collaborative IPC interventions to prevent the spread of CRKP in a teaching hospital in Shanghai.

## Methods

### Settings and ethics statement

The tertiary-level ICU of the Ruijin Hospital, Shanghai Jiao Tong University School of Medicine in Shanghai, consists of 12 beds including 8 private rooms and 2 double-occupancy rooms, and can hold approximately 180–250 critically ill patients annually. The study population consisted of all consecutive patients admitted to the ICU from 1 January 2013 through 30 June 2016. We employed a quasi-experimental before-and-after study design.

### Bacterial isolation, antimicrobial susceptibility testing, and clinical data collection

When patients were admitted to the ICU ward, pathogens screening had been carried out immediately and the second ASCs had been done within a week. Then routine and active surveillance cultures (ASCs) from various samples (nasopharyngeal swabs, sputum, endotracheal aspirate, urinary tract, and other possible infection sites) had been collected to monitor the incidence of CRKP colonisation/infection twice a week on Monday and Thursday. All isolates were identified using the VITEK2 compact system (bioMérieux, France), and routine antibiotic susceptibility tests were performed by the disk-diffusion assay to identify carbapenem resistance; susceptibility breakpoints were interpreted as per the Clinical and Laboratory Standards Institute guidelines [13]. Two specialists in infection biology helped collect patients’ clinical and microbiological data on colonisation and/or CRKP infection, including demographic characteristics, comorbid conditions, severity of illness, type of specimen, invasive procedures, antibiotic therapy, and outcomes. *Escherichia coli* ATCC 25922 was used as the quality-control strain.

We retrospectively analyzed the genetic relationships of the 18 collected CRKP isolates from October 2015 to June 2016 by carbapenemase genes (*bla*_KPC_, *bla*_IMP_, *bla*_NDM_, *bla*_VIM_, and *bla*_OXA-48_) sequencing, multilocus sequence typing (MLST) and pulsed-field gel electrophoresis (PFGE) methods to illustrate the main transmission clones in our ICU ward. Related methods were showed in Additional file 1 in details.

### Infection-control interventions and data collection

The study was implemented in 4 stages (Table 1). The first stage was a 6-month baseline period included patients who were admitted to the ICU during January 2013–June 2013, during which no intervention was performed and regular culture surveys were conducted to measure the prevalence of CRKP colonisation/infection.

**Table 1:** Interventions undertaken to curtail the epidemic spread of CRKP in 4 cumulative stages ^a^

Intervention	Description	Date begun
Baseline period	No intervention and regular surveillance cultures	Jan 2013- June 2013
Period 1	1. Active surveillance cultures 2. De-escalation interventions	July 2013–June 2014
	2.1 First level interventions	
	• contact precautions • patient isolation: single room isolation or cohorting • cohorting of medical care • disinfection and sterilization	
	2.2 Second level interventions	
	• contact precautions • disinfection and sterilization	
IPC interventions	2.3 Third level interventions	
	• disinfection and sterilization	
	3. Target bundles interventions	
	• intravascular catheter-related infection • ventilation associated pneumonia • catheter-associated urinary tract infection • skin and soft tissue infections.	
Period 2	1. Active surveillance cultures 2. Modified de-escalation interventions	July 2014–June 2015
Modified IPC interventions	+ Enhanced external medical staff education + Contact precautions of shared equipment + Enhanced terminal room disinfection	
	2.1 First level interventions (as Period 1) 2.2 Second level interventions (as Period 1) 2.3 Third level interventions (as Period 1)	
	3. Target bundles interventions (as Period 1)	
Period 3	Follow up period 2	July 2015–June 2016

^a^The procedures of infection control interventions are explicated in detail in the section of Materials and Methods

*IPC* Infection prevention and control, *CRKP* Carbapenem-resistant *Klebsiella pneumoniae*

**Fig. 1:**
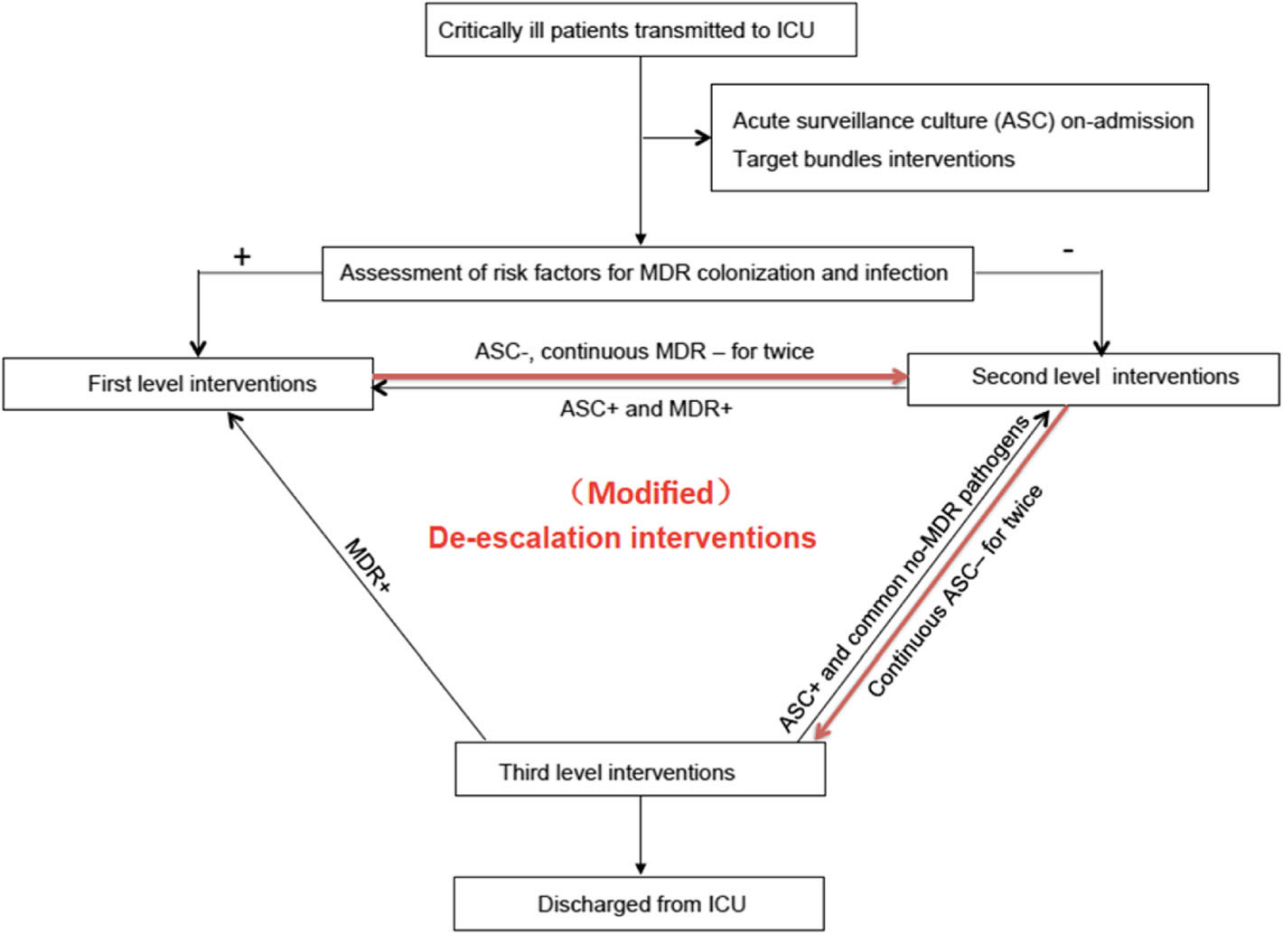
The procedures of (Modified) de-escalation interventions in period 2 and 3. Patients were active surveillance culture (ASC) for pathogens colonization and infection twice a week when admitted to the ICU, and assesse the risk factors of MDR bacteria colonization and infection immediately. For patients were defined infection/colonization with MDR pathogens, or came from clinical department with high prevalence of MDR bacteria before admitted to ICU, with invasive devices and damaged skin barrier such as endotracheal intubation, central venous catheter and urinary catheter etc., first level interventions measures were taken. For patients came from clinical department with low detection rate of MDR bacteria and just infected/colonized with common pathogens before admitted to ICU, second level interventions were taken. However, patients without bacteria infection/colonization or high risk factors, simple third level interventions were implemented. When patients in first level interventions have 2 consecutive negative tests which should be carried out over at least 1 week, descend to second level interventions. When continuous twice ASC negative results for second level patients, descend to third level interventions, and upgrade to first level interventions once MDR bacteria was detected during their ICU stay

The second stage—standard IPC intervention period including contact precautions, patient isolation, cohorting of medical care and disinfection and sterilization with de-escalation strategy and targeted bundle interventions aiming at intravascular catheter-related infection, ventilator-associated pneumonia, catheter-associated urinary tract infection and skin and soft tissue infections were implemented (Fig. 1)—during July 2013–June 2014. ASCs for pathogen colonisation/infection were performed twice a week, and the risk factors for MDR bacteria colonisation/infection were assessed [14]. First-level interventions were performed for patients who were diagnosed with MDR pathogen colonisation/infection or transferred from a clinical department with a high prevalence of MDR bacteria to the ICU and had invasive devices and skin-barrier damage by endotracheal intubation, central venous catheter insertion, etc. The first-level interventions included the following: (i) Contact precautions: adherence to hand-hygiene protocols before and after patient care and wearing gowns and gloves before patient care. (ii) Patient isolation: single-room isolation or cohorting. Patients with same MDR pathogens were admitted in double-occupancy rooms or a concentrated area of the unit, managed by dedicated nursing staff, and supplied with separate disposable medical equipment. (iii) Disinfection and sterilisation: To decrease the risk of transmission and burden of organisms, terminal disinfection of rooms was performed after patients been transferred or discharged out of ICU, different from ultraviolet irradiation disinfection regardless of time and distance in the baseline period. Environmental cleaning in the intervention period was enhanced including cleaning of areas in close proximity to the patient and irradiation with ultraviolet light from a close distance for at least 24 h after patients release. Additionally, targeted bundle interventions including those for intravascular catheter-related infection, ventilator-associated pneumonia, catheter-associated urinary tract infection, and skin and soft tissue infections were performed as previously described [15]. For patients who were transferred from a clinical department with a lower prevalence of MDR bacteria than the ICU or were colonised/infected with common pathogens before ICU admittance, second-level interventions including contact precautions, disinfection, and sterilisation (irradiation with ultraviolet light from a close distance for at least 2 h) were implemented. For patients without bacterial colonisation/infection or high-risk factors, third-level interventions including hand hygiene, disinfection, and sterilisation measures were used. During the study, when patients receiving first-level interventions had 2 consecutive negative ASCs which should be carried out over at least 1 week, they were de-escalated to second-level interventions. When second-level patients showed 2 consecutive negative ASCs, they were de-escalated to third-level interventions. Patients in any level were upgraded to first-level interventions if MDR bacteria were detected during their ICU stay.

The third stage—modified IPC intervention period—during July 2014–June 2015. Modified IPC interventions, in addition to the standard IPC interventions, which included extensive external medical staff education (all consulting staff, rehabilitative physicians, and external nurses should receive lecture and practices on infection prevention education once a month, including the importance and procedures of infection control measures); extensive contact precautions and cleaning of shared equipment (radiography and ultrasound machines); and extensive terminal room disinfection, especially for areas in close proximity to CRKP carriers such as surfaces around sinks. Once the CRKP patients were discharged, extensive terminal cleaning of CRKP patient rooms and monitoring of the cleaning process were performed to ensure that all surfaces were adequately cleaned and disinfected.

The fourth stage—follow-up period—involved a follow-up in addition to the modified IPC interventions during July 2015–June 2016.

### Definitions

ICU-acquired infection was confirmed when pathogens were not present at the time of admission, but detected by ASC after ICU admission for > 48 h [16]. Isolates not susceptible to imipenem, meropenem, or ertapenem were considered carbapenem-resistant. Central line-associated bloodstream infection, ventilator-associated pneumonia, and catheter-associated urinary tract infection were defined as per the Centers for Disease Control guidelines [15, 17–19].

The incidence of CRKP colonisations/infections detected by cultures was measured and standardised to the number of cases per 1000 ICU patient-days according to the Centers for Disease Control criteria [14]. The monthly incidence of CRKP positivity was calculated. CRKP colonisation/infection was measured and compared with the overall periodic incidence. The primary endpoint was the monthly incidence of ICU-acquired CRKP patients (no. of cases/1000 ICU patient-days) during the baseline and different intervention periods. Subsequently, the incidence of infections form different sites (no. of cases per 1000 catheter-days/ventilator-days /ICU patient-days) was calculated.

### Statistical analysis

All statistical tests were performed using SPSS software, version 17.0 (Chicago, IL, USA). Discrete variables were summarised as frequency (%) and continuous variables, as mean and standard deviation or median and interquartile range. Student’s *t*-test and the Mann-Whitney *U*-test were used to compare continuous variables. Continuous variables were compared among multiple groups using variance analysis and Students-Newman-Keuls test (normal distribution and equal variances assumed) or rank sum test (non-normal distribution or equal variances not assumed). Categorical variables were compared using the χ^2^ test or Fisher’s test, as appropriate. All tests were 2-tailed, and values of *P <* 0.05 were considered statistically significant. Segmented (interrupted) linear regression (Additional file 1: Table S2) was performed to examine whether the use of predetermined IPC interventions affected incidence.

## Results

A total of 629 patients were enrolled during the entire study: 74 cases in the baseline period, 187 cases in the standard IPC intervention period, 222 cases in the modified IPC intervention period, and 146 cases in the follow-up period. Additionally, 87 patients had CRKP, of which 69 (79.3%) patients acquired the infection in the ICU and 18 (20.7%) acquired the infection prior to ICU admission. Patient data such as general characteristics, presence of chronic disease, organ failure, risk factors of CRKP colonisation/infection were described in Table 2. The Acute Physiology and Chronic Health Evaluation II score on ICU admission, proportion of patients with organ failure such as congestive heart failure and acute respiratory failure, and exposure to carbapenems before CRKP colonisation/infection were higher during the intervention periods as compared to the baseline. However, the number of invasive operations such as tracheal intubation, surgeries, central line catheter insertions, and indwelling urinary catheter insertions before CRKP colonisation/infection were lower in the follow-up period than in the intervention periods. All other patient characteristics during the ICU stay were similar among the 4 study periods.

**Table 2:** Clinical characteristics of the ICU patients, according to study period

Variable	Baseline period	Period 1	Period 2	Period 3	*P* value
General characteristics					
Patients (no.)	74	187	222	146	
Age (yr), mean ± SD	63 ± 17	62 ± 18	61 ± 18	63 ± 19	0.869
Male sex (%)	52(70.3%)	114(61%)	134(60.4%)	90(61.6%)	0.476
APACHEII score, mean ± SD	15 ± 7	15 ± 7	16 ± 8	19 ± 9	0.001
Chronic disease					
Malignancy(%)	29(39.2%)	90(48.1%)	108(48.6%)	59(40.4%)	0.249
Chronic obstructive pulmonary disease(%)	3(4.1%)	6(3.2%)	13(5.9%)	8(5.5%)	0.591
Diabetes(%)	12(16.2%)	24(12.8%)	35(15.8%)	20(13.7%)	0.811
Hypertension(%)	14(18.9%)	55(29.4%)	60(27%)	54(37%)	0.035
Coronary heart disease(%)	5(6.8%)	10(5.3%)	19(8.6%)	7(4.8%)	0.454
Organ failure					
Congestive heart failure(%)	4(5.4%)	8(4.3%)	26(11. 7%)	19(13%)	0.011
Acute respiratory failure(%)	12(16.2%)	51(27.3%)	72(32.4%)	66(45.2%)	*P* < 0.001
Acute renal injury(%)	16(21.6%)	41(21.9%)	50(22.5%)	46(31.5%)	0.147
Acute gastrointestinal injury (%)	5(6.80%)	25(13.4%)	25(11.3%)	33(22.6%)	0.003
Hepatic insufficiency(%)	6(8.1%)	20(10.7%)	26(11.7%)	26(17.8%)	0.123
Exposure to antimicrobial therapy before CRKP colonization/infection					
Carbapenem	44(59.5%)	99(52.9%)	115(51.8%)	94(64.4%)	0.078
Cephalosporin antibiotics	56(75.7%)	135(72.7%)	177(79.7%)	94(64.4%)	0.012
Sulbactam/ cefoperazone	12(16.2%)	29(15.5%)	43(19.4%)	27(18.5%)	0.749
Mechanical ventilation before CRKP colonization/infection					
Tracheal intubation	63(85.1%)	162(86.6%)	197(88.7)	110(75.3%)	0.004
Tracheotomy	11(4.9%)	22(11.8%)	23(10.8%)	25(17.1%)	0.318
Days of mechanical ventilation, days, mean ± SD	7 ± 14	7 ± 20	5 ± 14	10 ± 23	0.104
Surgery or invasive devices before CRKP colonization/infection					
Surgery	61(82.4%)	160(85.6%)	179(80.6%)	103(70.5%)	0.007
Central line catheter	71(95.9%)	165(88.2%)	180(81.1%)	113(72.4%)	P < 0.001
Thoracentesis	9(12.2%)	17(9.1%)	23(10.4%)	24(16.4%)	0.184
Abdominal paracentesis	5(6.8%)	25(13.4%)	21(9.5%)	18(12.3%)	0.356
Continuous renal replacement therapy	1(1.4%)	26(13.9%)	22(9.9%)	19(13%)	0.022
Indwelling urinary catheter	70(94.6%)	182(97.3%)	218(98.2%)	135(92.5%)	0.027

The microbiological characteristics and genetic relatedships of the 18 collected CRKP isolates from October 2015 to June 2016 were retrospectively analyzed. 13 *bla*_KPC_ positive, 3 *bla*_VIM_ positive and one *bla*_OXA_-48 positive CRKP isolates were detected. MLST data showed that these 13 KPC-2 carbapenemases producing CRKP isolates belonged to the ST11 group and that their PFGE patterns were highly similar between on-admission and ICU-acquired CRKP isolates. (Additional file 1: Table S1 and Figure S1). However, genome of detected K. pneumonia should be sequenced and compared to clarify the main transmission clones of ICU-acquired CRKP in our future infection control measures and studies.

**Fig. 2:**
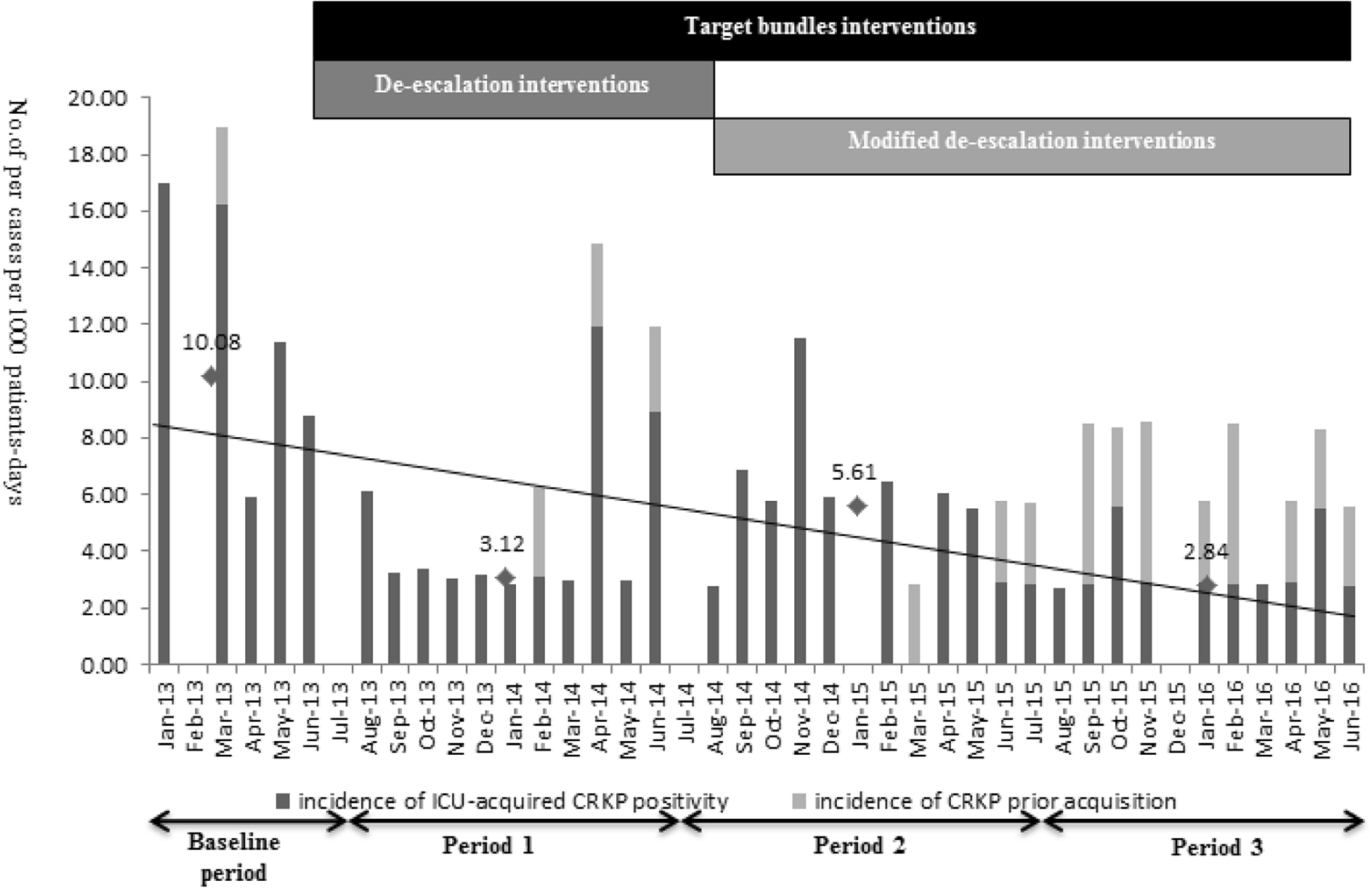
Epidemic of observed monthly incidence rate of patients (No. of cases per 1000 ICU patient-days) colonized or infected with CRKP in the intensive care unit during the baseline and different intervention periods. Black bars show the incidence rate of ICU-acquired CRKP positivity, and gray bars represent the incidence rate of CRKP patients at ICU admission surveillance. Trend lines of ICU-acquired CRKP (No. of cases per 1000 ICU patient-days) for each period are shown in black solid bold type, showed a downward incidence rate trend from 10.08 to 2.84 after the implement of modified IPC interventions (P<0.05)

The monthly incidence of totally CRKP colonisation/infections including ICU-acquired CRKP and CRKP acquisition prior ICU admission were showed in Fig. 2 and Table 3. After the modified IPC intervention, a significant reduction in the overall incidence was observed (Table 3). The monthly incidence of ICU-acquired CRKP was 10.08 (4.43–16.43) cases per 1000 ICU patient-days at the baseline, and IPC interventions were associated with a clear significant decrease to 3.12 (2.98–5.40), but was followed by several increases in the incidence in April 2014(Fig. 2). The prevalence rates stabilised and decreased with implementation of the modified IPC interventions in the modified IPC intervention (5.62 (0.69–6.34)) and follow-up periods (2.84 (2.80–2.89)) as compared to the baseline period (*P* = 0.032 and 0.021). There was no significant difference in the monthly incidence rates between the standard and modified IPC intervention periods. Although patients who acquired infections prior to ICU admission showed an increasing distribution during the follow-up period, there was a continuous declination in the incidence of ICU-acquired CRKP infections with respect to compliance with modified IPC interventions, (P = 0.032). Segmented (interrupted) linear regression was added to examine whether the use of predetermined IPC interventions affected incidence. IPC interventions period did not showed a significant change in incidence due to short time of baseline period. However, compared with IPC interventions period, the success of modified IPC interventions was shown by the negative slope value which was associated with continuous declination of monthly incidence of ICU-acquired CRKP colonization/ infection (*p* = 0.036). Results were shown in Additional file 1: Table S2.

**Table 3:** CRKP distribution and the incidence of ICU-acquired infections from different infection sites

Variable	Baseline period	Period 1	Period 2	Period 3	p
No. cases of total CRKP	22	20	20	25	
No. cases of ICU-on-admission CRKP	1	3	2	12	
No. cases of ICU-acquired CRKP	21	17	18	13	
Probable source of colonization/ infection of ICU-acquired CRKP					
Bloodstream n(%)	3/21(14.3)	6/17(35.3)	1/18(5.6)	1/13(7.7)	0.11
Respiratory tract n(%)	4/21(19)	3/17(17.6)	5/18(27.8)	5/13(38.5)	0.555
Skin and soft tissue n(%)	13/21(61.9)	7/17(41.2)	12/18(66.7)	3/13(23.1)	0.058
Urine tract n(%)	1/21(4.8)	1/17(5.9)	0/18(0)	4/13(25)	0.073
Incidence-ICU acquired					
Incidence of CRBSI (No. of cases per 1000 catheter days)	2.54	2.44	0.43	0.41	0.352
Incidence of VAP (No. of cases per 1000 ventilator-days)	2.84	0.48	0.49	0.81	0.41
Incidence of skin and soft tissue infections (No. of cases per 1000 ICU patient-days)	3.40	0.99	1.46	0.47	0.531
Incidence of catheter-associated urinary tract infection (No. of cases per 1000 catheter days)	0	0	0	0.34	0.63

During the entire study, the probable source of colonisation/infection of ICU-acquired CRKP was the bloodstream, respiratory tract, skin and soft tissues, and urinary tract (Table 4). The incidence of ICU-acquired CRKP catheter-related bloodstream infections per 1000 central line catheter-days decreased from 2.54 during the baseline period to 0.41 during the follow-up period, although the difference was not statistically significant. Additionally, there was a downward trend in the incidence of ICU-acquired CRKP ventilator-associated pneumonia and skin and soft tissue infections from 2.84 to 0.41 infections per 1000 ventilator-days and from 3.4 to 0.47 infections per 1000 ICU patient-days, with slight fluctuations in the trend. In contrast, there was no difference in the incidence of urinary tract infections.

**Table 4:** Monthly incidence rates of totally CRKP colonization/ infection and ICU-acquired CRKP (No. of cases per 1000 ICU patient-days)

Intervention (period)	Incidence				Incidence			
	No. of cases per 1000 ICU patient-days (totally CRKP)				No. of cases per 1000 ICU patient-days (ICU-acquired CRKP)			
	Mean	Median	P^a^	P^b^	Mean	Median	P^c^	P^d^
Baseline period	10.33 ± 7.05	10.08 (4.43–17.46)	/	/	9.88 ± 6.44	10.08 (4.43–16.43)	/	/
Period 1	5.05 ± 4.25	3.18 (2.98–6.18)	0.014	/	4.30 ± 3.2	3.12 (2.98–5.40)	0.024	/
Period 2	4.94 ± 3.18	5.76 (2.77–6.34	0.013	0.945	4.47 ± 3.46	5.62 (0.69–6.34)	0.032	0.9
Period 3	5.88 ± 2.81	5.78 (3.52–8.47)	0.037	0.577	3.05 ± 1.42	2.84 (2.80–2.89)	0.021	0.032

^a^Compared with baseline period

^b^Compared with period 1

^c^Compared with baseline period

^d^Compared with period 1

## Discussion

This report describes the management of an outbreak of CRKP colonisation/infection until final control of the epidemic and includes clinical data of > 600 ICU patients over a period of almost 4 years. Implementation of an infection-prevention bundle and control interventions led to clinically important and statistically significant decreases in ICU-acquired CRKP colonisations/infections at a tertiary-level ICU in a developing country. To our knowledge, this is one of the first few comprehensive IPC studies including de-escalation and targeted bundle interventions that aimed at reducing the incidence of ICU-acquired CRKP in China.

Thus far, there are no universally useful and successful infection-control measures in hospital settings in low- and middle-income countries. The current medical literature does not provide sufficient data to determine which model of infection control is the most appropriate and effective in controlling the spread of CRKP in the ICU [20, 21]. Our proposed intervention comprises 3 main components: ASC; de-escalation interventions; and targeted bundle interventions. Although the efficacy of ASC has been described previously [22, 23], our study showed that ASC allowed early identification of CRKP carriers and guided de-escalation interventions to stop contact precautions and initiate patient isolation in a timely manner. Through this approach, patients with different risk factors were provided hierarchical prevention and control measures and were upgraded or de-escalated according to the ASC results. A 4-year perspective study in Israel [10] showed that isolation precaution alone was ineffective. Our bundled IPC interventions, including isolation precaution, showed a rapid decrease in the incidence of ICU-acquired CRKP colonisations/infections. In addition to de-escalation interventions, targeted bundles led to a downward trend in the incidence of ICU-acquired CRKP infections including central line-associated bloodstream infection, ventilator-associated pneumonia, and skin and soft tissue infections.

When carbapenem-resistant pathogen outbreaks occurred with multimodal interventions, it was difficult to determine which of the interventions impacted the transmission of CRKP. A study on carbapenem-resistant *Acinetobacter baumannii* outbreak in an Italian ICU [24] showed that medical staff should focus on standard contact precautions, especially hand-washing with alcohol-based solutions and personal protective equipment. In contrast, a 5-year Greek study [25] suggested that the most-influential factors responsible for a decrease in the incidence of carbapenem-resistant pathogen in the bloodstream infections were increased participation in educational courses. Feedback from screening services, rapid turnaround time and efficient communication were correlated with overall institutional success in outbreak control [26, 27]. In our study, although de-escalation and targeted bundle interventions helped to reduce the incidence of CRKP during IPC interventions, the reappearance of ICU-acquired CRKP in April 2014 suggested that additional factors were involved. Therefore, the modified IPC interventions were implemented according to the monitoring and analysis of screening results and proved successful in preventing the transmission of CRKP with prompt implementation of organizational measures such as non-ICU medical staff education, contact precautions for shared equipment, and multiple rigorous cleaning and disinfection interventions. The incidence of ICU-acquired CRKP colonisations and infections continued to decrease despite the extremely high number of cases of previous CRKP acquisition at ICU admission in period 2 and 3, suggesting that these measures may contributed to decrease the incidence of ICU-acquired infections. The integration of epidemiological and microbiological data and the strict application of infection-control measures played a decisive role in preventing against the spread of CRKP in our hospital.

In developing countries, shortage of medical supplies is known to contribute to a low adherence to adequate IPC practices and increase the risk of nosocomial infections [25]. The follow-up period is more important than the other periods, as it involves evaluation of the effectiveness and practicality of IPC interventions during a prolonged national outbreak [28, 29]. In a 1-year follow-up period, the incidence of ICU-acquired CRKP colonisations and infections continued to decrease despite the extremely high number of cases of previous CRKP acquisition at ICU admission.

During the entire study period, we implemented a de-escalation antimicrobial therapy based on culture results and the clinical status of patients. But the impact of de-escalation antimicrobial therapy on antimicrobial resistance was not analyzed. While other studies have shown a relationship between antimicrobial use and resistance. Antimicrobial stewardship programs especially de-escalation antimicrobial therapy has been shown to improve patient outcomes, reduce antimicrobial adverse events, and decrease antimicrobial resistance [30, 31].

Our study had a few limitations. Firstly, this was a single-centre study; therefore, the number of included patients was relatively small and the results may not be generalizable to other institutions. Further multicentre, prospective studies are needed to confirm our findings. Secondly, the interventions described in this study were multimodal due to clinical necessity, which precludes determination of the effectiveness of any single measure. Thirdly, compliance with various interventions during the study period was not assessed.

## Conclusion

In summary, comprehensive IPC interventions including modified de-escalation and targeted bundle interventions played a pivotal role in controlling the epidemic spread of ICU-acquired CRKP colonisations/infections in our hospital in China.

## Additional file

Additional file 1: **Table S1.** The microbiological characteristics and genetic relatedness of the 18 collected CRKP isolates. **Table S2.** Central distribution values and regression slopes for the monthly incidence of colonization/Infection with ICU-acquired CRKP (No. of cases per 1000 ICU patient-days). **Figure S1.** Pulsed-field gel electrophoresis (PFGE) of XbaI-digested DNA of 13 KPC-2 producing CRKP isolates. PFGE marker, Salmonella enterica H9812. (DOCX 651 kb)

## Abbreviations

ASC: Active surveillance cultures
CRKP: Carbapenem-resistant *Klebsiella pneumonia*
ICU: Intensive care unit
IPC: Infection-prevention and control
MDR: Multidrug-resistant

## References

[1] Bassetti M, Poulakou G, Ruppe E, Bouza E, Van Hal SJ, Brink A (2017). Antimicrobial resistance in the next 30 years, humankind, bugs and drugs: a visionary approach. Intensive Care Med.

[2] Guan X, He L, Hu B, Hu J, Huang X, Lai G (2016). Laboratory diagnosis, clinical management and infection control of the infections caused by extensively drug-resistant gram-negative bacilli: a Chinese consensus statement. Clin Microbiol Infect.

[3] Tian L, Tan R, Chen Y, Sun J, Liu J, Qu H (2016). Epidemiology of *Klebsiella pneumoniae* bloodstream infections in a teaching hospital: factors related to the carbapenem resistance and patient mortality. Antimicrob Resist Infect Control.

[4] Friedman ND, Carmeli Y, Walton AL, Schwaber MJ (2017). Carbapenem-resistant Enterobacteriaceae: a strategic roadmap for infection control. Infect Control Hosp Epidemiol.

[5] Solomon SL, Oliver KB (2014). Antibiotic resistance threats in the United States: stepping back from the brink. Am Fam Physician.

[6] Xu Y, Gu B, Huang M, Liu H, Xu T, Xia W (2015). Epidemiology of carbapenem resistant Enterobacteriaceae (CRE) during 2000-2012 in Asia. J Thorac Dis.

[7] Van Duijn PJ, Verbrugghe W, Jorens PG, Spohr F, Schedler D, Deja M (2018). The effects of antibiotic cycling and mixing on antibiotic resistance in intensive care units: a cluster-randomised crossover trial. Lancet Infect Dis.

[8] Hayden MK, Lin MY, Lolans K, Weiner S, Blom D, Moore NM (2015). Prevention of colonization and infection by *Klebsiella pneumoniae* carbapenemase-producing enterobacteriaceae in long-term acute-care hospitals. Clin Infect Dis.

[9] Van Loon K, Voor In’t Holt AF, Vos MC. A Systematic Review and Meta-analyses of the Clinical Epidemiology of Carbapenem-Resistant Enterobacteriaceae. Antimicrob Agents Chemother. 2017;62(1). 10.1128/AAC.01730-17. 10.1128/AAC.01730-17 PMC5740327 29038269

[10] Cohen MJ, Block C, Levin PD, Schwartz C, Gross I, Weiss Y (2011). Institutional control measures to curtail the epidemic spread of carbapenem-resistant *Klebsiella pneumoniae*: a 4-year perspective. Infect Control Hosp Epidemiol.

[11] Laurent C, Rodriguez-Villalobos H, Rost F, Strale H, Vincent JL, Deplano A (2008). Intensive care unit outbreak of extended-spectrum beta-lactamase-producing *Klebsiella pneumoniae* controlled by cohorting patients and reinforcing infection control measures. Infect Control Hosp Epidemiol.

[12] Borer A, Eskira S, Nativ R, Saidel-Odes L, Riesenberg K, Livshiz-Riven I (2011). A multifaceted intervention strategy for eradication of a hospital-wide outbreak caused by carbapenem-resistant *Klebsiella pneumoniae* in southern Israel. Infect Control Hosp Epidemiol.

[13] Zhang F, Wang X, Xie L, Zheng Q, Guo X, Han L (2017). A novel transposon, Tn6306, mediates the spread of blaIMI in Enterobacteriaceae in hospitals. Int J Infect Dis.

[14] Magiorakos AP, Burns K, Rodriguez Bano J, Borg M, Daikos G, Dumpis U (2017). Infection prevention and control measures and tools for the prevention of entry of carbapenem-resistant Enterobacteriaceae into healthcare settings: guidance from the European Centre for Disease Prevention and Control. Antimicrob Resist Infect Control.

[15] Krein SL, Greene MT, Apisarnthanarak A, Sakamoto F, Tokuda Y, Sakihama T (2017). Infection prevention practices in Japan, Thailand, and the United States: results from National Surveys. Clin Infect Dis.

[16] Lopez-Pueyo MJ, Olaechea-Astigarraga P, Palomar-Martinez M, Insausti-Ordenana J, Alvarez-Lerma F (2013). Quality control of the surveillance programme of ICU-acquired infection (ENVIN-HELICS registry) in Spain. J Hosp Infect.

[17] Lo E, Nicolle LE, Coffin SE, Gould C, Maragakis LL, Meddings J (2014). Strategies to prevent catheter-associated urinary tract infections in acute care hospitals: 2014 update. Infect Control Hosp Epidemiol.

[18] Marschall J, Mermel LA, Fakih M, Hadaway L, Kallen A, O'Grady NP (2014). Strategies to prevent central line-associated bloodstream infections in acute care hospitals: 2014 update. Infect Control Hosp Epidemiol.

[19] Klompas M, Branson R, Eichenwald EC, Greene LR, Howell MD, Lee G (2014). Strategies to prevent ventilator-associated pneumonia in acute care hospitals: 2014 update. Infect Control Hosp Epidemiol.

[20] Bassetti M, Giacobbe DR, Giamarellou H, Viscoli C, Daikos GL, Dimopoulos G (2018). Management of KPC-producing Klebsiella pneumoniae infections. Clin Microbiol Infect.

[21] Tacconelli E, Cataldo MA, Dancer SJ, De Angelis G, Falcone M, Frank U (2014). ESCMID guidelines for the management of the infection control measures to reduce transmission of multidrug-resistant gram-negative bacteria in hospitalized patients. Clin Microbiol Infect.

[22] Gagliotti C, Cappelli V, Carretto E, Marchi M, Pan A, Ragni P, et al. Control of carbapenemase-producing *Klebsiella pneumoniae*: a region-wide intervention. Euro Surveill. 2014;19(43). 10.2807/1560-7917.es2014.19.43.20943 25375901

[23] Langer AJ, Lafaro P, Genese CA, McDonough P, Nahass R, Robertson C (2009). Using active microbiologic surveillance and enhanced infection control measures to control an outbreak of health care-associated extended-spectrum beta-lactamase-producing *Klebsiella pneumoniae* infections--New Jersey, 2007. Am J Infect Control.

[24] Bianco A, Quirino A, Giordano M, Marano V, Rizzo C, Liberto MC (2016). Control of carbapenem-resistant *Acinetobacter baumannii* outbreak in an intensive care unit of a teaching hospital in southern Italy. BMC Infect Dis.

[25] Kousouli E, Zarkotou O, Politi L, Polimeri K, Vrioni G, Themeli-Digalaki K (2018). Infection control interventions affected by resource shortages: impact on the incidence of bacteremias caused by carbapenem-resistant pathogens. Eur J Clin Microbiol Infect Dis.

[26] Lyles RD, Moore NM, Weiner SB, Sikka M, Lin MY, Weinstein RA (2014). Understanding staff perceptions about Klebsiella pneumoniae carbapenemase-producing Enterobacteriaceae control efforts in Chicago long-term acute care hospitals. Infect Control Hosp Epidemiol.

[27] Apisarnthanarak A, Ratz D, Khawcharoenporn T, Patel PK, Weber DJ, Saint S (2017). National Survey of practices to prevent methicillin-resistant *Staphylococcus aureus* and multidrug-resistant *Acinetobacter baumannii* in Thailand. Clin Infect Dis.

[28] Zhang Y, Wang Q, Yin Y, Chen H, Jin L, Gu B, et al. Epidemiology of Carbapenem-Resistant Enterobacteriaceae Infections: Report from the China CRE Network. Antimicrob Agents Chemother. 2018;62(2). 10.1128/AAC.01882-17. 10.1128/AAC.01882-17 PMC5786810 29203488

[29] Gurieva T, Dautzenberg MJD, Gniadkowski M, Derde LPG, Bonten MJM, Bootsma MCJ (2018). The transmissibility of antibiotic-resistant Enterobacteriaceae in intensive care units. Clin Infect Dis.

[30] Kelly AA, Jones MM, Echevarria KL, Kralovic SM, Samore MH, Goetz MB (2017). A report of the efforts of the veterans health administration national antimicrobial stewardship initiative. Infect Control Hosp Epidemiol.

[31] Dodds Ashley ES, Kaye KS, DePestel DD, Hermsen ED (2014). Antimicrobial stewardship: philosophy versus practice. Clin Infect Dis.

