# Supplementary material for: Infection-prevention and control interventions to reduce colonisation and infection of intensive care unit-acquired carbapenem-resistant Klebsiella pneumoniae: a 4-year quasi-experimental before-and-after study
Source: Antimicrob Resist Infect Control. 2019 Jan 10;8:8. doi: 10.1186/s13756-018-0453-7 (PMC6329090; doi:10.1186/s13756-018-0453-7)

**Infection-prevention and control interventions to reduce colonisation and infection of intensive care unit-acquired carbapenem-resistant *Klebsiella pneumoniae*: a 4-year quasi-experimental before-and-after study**

Meiling Li ^a,†^, Xiaoli Wang ^a,†^, Jiahui Wang ^a^ , Ruoming Tan ^a^, Jingyong Sun ^b^, Lei Li ^a^, Jie Huang ^a^, Jun Wu ^a^, Qiuying Gu ^a^, Yujin Zhao ^a^, Jialin Liu ^a,∗^ and Hongping Qu ^a, ∗^

^a^Department of Critical Care Medicine, Ruijin Hospital, Shanghai Jiao Tong University School of Medicine, No.197 Ruijin ER Road, Shanghai 200025, China

^b^Department of Clinical Microbiology, Ruijin Hospital, Shanghai Jiao Tong University School of Medicine, No.197 Ruijin ER Road, Shanghai 200025, China

^∗^ Correspondence: ljl11243@rjh.com.cn; hongpingqu0412@hotmail.com

† Meiling Li and Xiaoli Wang contributed equally to this work.

**Supplementary method**

**Carbapenemase genes sequencing, multilocus sequence typing (MLST) and pulsed-field gel electrophoresis (PFGE)**

Key carbapenemase genes (*bla*_KPC_, *bla*_IMP_, *bla*_NDM_, *bla*_VIM_, and *bla*_OXA-48_) were detected by polymerase chain reaction (PCR), using previously described primers.(Nordmann P, Naas T, Poirel L. Global spread of Carbapenemase-producing Enterobacteriaceae. Emerg Infect Dis 2011;17:1791–8.).The PCR products were purified and sequenced twice, bidirectionally, at Sangon Biotech (Shanghai, China). MLST of KPC-2 carbapenemases producing CRKP was performed according to the guidelines given on the *K. pneumoniae* MLST website (http:// [www.pasteur.fr/recherche/](http://www.pasteur.fr/recherche/)genopole/PF8/mlst/Kpneumoniae.html). Chromosomal DNA was prepared in agarose blocks and digested with XbaI. DNA fragments were separated by PFGE on a CHEF Mapper XA (Bio-Rad, Hercules, CA, USA) for 20 h at 14℃, at 6 V/cm, a pulse angle of 120°, and pulse times ranging from 2.16 to 54.17 s.

**Supplementary data**

**Table S1**. The microbiological characteristics and genetic relatedness of the 18 collected CRKP isolates.

| **CRKP isolate** | **source** | **Collection**  **date** | **ICU-on-**  **admission CRKP** | **ICU-acquired CRKP** | **KPC** | **OXA** | **VIM** | **MLST** | **PFGE** |
| --- | --- | --- | --- | --- | --- | --- | --- | --- | --- |
| **286** | Skin and soft tissue | Oct,2015 | + |  |  |  | + |  |  |
| **8135** | Respiratory tract | Oct,2015 |  | + | + |  |  | ST11 | A2 |
| **B214** | Urine tract | Nov,2015 | + |  |  |  | + |  |  |
| **B547** | Urine tract | Nov,2015 | + |  | + |  |  | ST11 | A |
| **B756** | Urine tract | Nov,2015 |  | + | + |  |  | ST11 | A2 |
| **B436** | Urine tract | Jan,2016 | + |  | + |  |  | ST11 | A1 |
| **8273** | Respiratory tract | Jan,2016 |  | + | + |  |  | ST11 | A2 |
| **1568** | Respiratory tract | Feb,2016 | + |  |  |  | + |  |  |
| **124** | Respiratory tract | Feb,2016 | + |  | + |  |  | ST11 | A |
| **1125** | Respiratory tract | Feb,2016 |  | + | + |  |  | ST11 | A1 |
| **1420** | Skin and soft tissue | Mar,2016 |  | + | + |  |  | ST11 | A2 |
| **8520** | Respiratory tract | Apr,2016 | + |  | + |  |  | ST11 | A |
| **1792** | Skin and soft tissue | Apr,2016 |  | + | + |  |  | ST11 | A2 |
| **8382** | Respiratory tract | May,2016 | + |  | + |  |  | ST11 | A1 |
| **879** | Skin and soft tissue | May,2016 |  | + | + |  |  | ST11 | A1 |
| **8700** | Respiratory tract | May,2016 |  | + |  |  |  |  |  |
| **8379** | Respiratory tract | Jun,2016 |  | + |  | + |  |  |  |
| **1324** | Skin and soft tissue | Jun,2016 | + |  | + |  |  | ST11 | A |

**Table S2**. Central distribution values and regression slopes for the monthly incidence of colonization/Infection with ICU-acquired CRKP （No. of cases per 1,000 ICU patient-days）

| **Period** | **Median incidence** | **Slope^a^ 95% CI** | ***P* value^b^** |
| --- | --- | --- | --- |
| **Baseline**  **(Jan 2013-June 2013)** | 10.08(4.43-16.43) | -0.487(-5.219，4.246) | / |
| **IPC interventions**  **(July 2013-June2014)** | 3.12(2.98-5.40) | 0.451(-0.089，0.991) | 0.428 |
| **Modified IPC interventions**  **(July 2014-June 2016)** | 2.87(2.76-5.71) | -0.083(-0.247，0.081) | 0.036 |

^a^Linear regression model coefficient.

^b^The probability that the change in slope, compared with the slope of the previous period

**Figure S1**. Pulsed-field gel electrophoresis (PFGE) of XbaI-digested DNA of 13 KPC-2 producing CRKP isolates. PFGE marker, Salmonella enterica H9812.


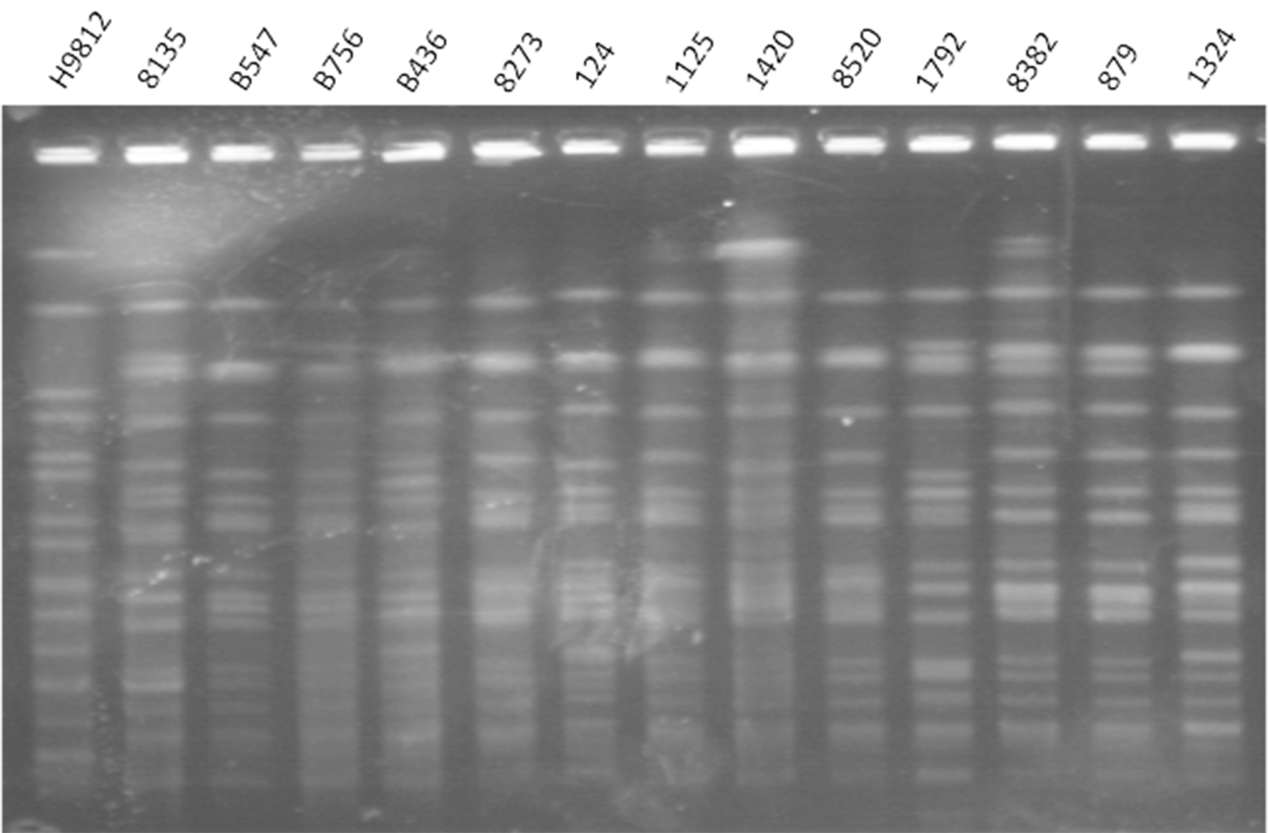

Supplement: Supplementary file 1 — Table S1. The microbiological characteristics and genetic relatedness of the 18 collected CRKP isolates. Table S2. Central distribution values and regression slopes for the monthly incidence of colonization/Infection with ICU-acquired CRKP (No. of cases per 1000 ICU patient-days). Figure S1. Pulsed-field gel electrophoresis (PFGE) of XbaI-digested DNA of 13 KPC-2 producing CRKP isolates. PFGE marker, Salmonella enterica H9812. (DOCX 651 kb) [file 13756_2018_453_MOESM1_ESM.docx]
